# Behaviour of Brown Bears Under Fluctuating Resource Availability

**DOI:** 10.1002/ece3.71693

**Published:** 2025-06-27

**Authors:** Clara Tattoni, Andrea Corradini, Francesco Chianucci, Marco Ciolli, Roberta Giusti, Natalia Bragalanti, Francesca Cagnacci, Adriano Martinoli, Damiano G. Preatoni, Francesco Bisi

**Affiliations:** ^1^ Dipartimento di Scienze Teoriche e Applicate Università degli Studi dell'Insubria Varese Italy; ^2^ Fondazione Edmund Mach San Michele all'Adige Italy; ^3^ CREA, Research Centre for Forestry and Wood Arezzo Italy; ^4^ DICAM – Università di Trento Trento Italy; ^5^ C3A – Centro Agricoltura Alimenti Ambiente San Michele all'Adige Italy; ^6^ Ufficio foreste e fauna Provincia Autonoma di Trento Trento Italy

**Keywords:** Alps, beech mast, damage, home range, MaxEnt modelling, *Ursus arctos*

## Abstract

Mast seeding, the variable and intermittent production of seeds, has cascading effects on ecosystem functioning. This study explores its influence on the brown bear populations in the Italian Alps, focusing on beechnuts ( *Fagus sylvatica* L.), the primary food source for bears in the region. Using historical data and field sampling, we estimated and mapped the annual seed biomass from 2007 to 2021 for the province of Trento. The energy content of beechnuts was assessed through high heating values, providing the caloric resources available. Data on beechnuts production, records of damages and GPS data from 16 Eurasian brown bears were integrated to perform a temporal and spatial analysis at home range and at landscape level. Standardised damages to beehives and livestock decreased during mast years, suggesting that bears met their trophic needs through natural food sources. In fact, bears used more agricultural areas and less beech forest during years of beech crop failure. At landscape level, agriculture and pasture areas close to beech forests and distant from cities showed a higher risk of damage, providing a tool to anticipate management actions. This work provides insights on the ecological dynamics and conservation implications of brown bears in the study area by mapping the spatial and temporal aspects of mast seeding and bear‐related damages.

## Graphical abstract

Using a 20‐year period of observations from different sources, this article examines the spatial and temporal patterns of beech masting in relation to brown bear movement and damage to livestock and human property. Standardised damages to beehive and livestock decreased during mast years, suggesting that bears met their trophic needs through natural food sources.

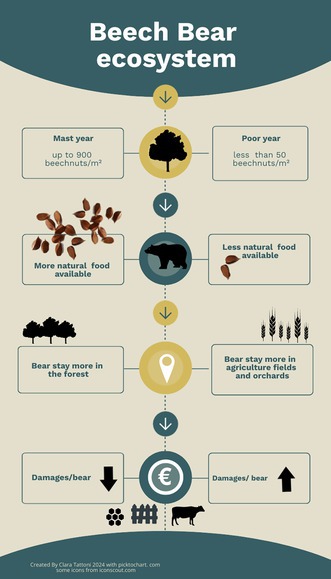

## 1 Introduction

Mast seeding is the variable and intermittent production of large seed crops, which is a typical reproductive strategy of many wind‐pollinated plant species (Kelly 1994; Bogdziewicz et al. 2024). Masting events have a cascading effect on the functioning of the ecosystem as a whole; for example, peaks in seed crops are relevant to the population dynamics of seed consumers such as rodents (Elkinton et al. 1996; Zwolak et al. 2016; Franzoi Dri et al. 2024), roe deer and wild boar (Canu et al. 2015; Bisi et al. 2016, 2018; Gamelon et al. 2021), brown bear (Ciucci et al. 2014; Tattoni et al. 2015; McClelland et al. 2020), many birds and insects (Tattoni, Chianucci, et al. 2019; Tattoni, Soardi, et al. 2019; Bogdziewicz et al. 2018) and reverberate across trophic levels (Czeszczewik et al. 2020; Bogdziewicz et al. 2016).

Masting creates cycles of feast and famine in food webs. These cycles strongly influence the behaviour and life‐history strategies of consumers (Zwolak et al. 2016). For instance, periodic food failure was indicated as one of the main drivers of human‐bear conflict globally, according to a survey addressed to field biologists and managers, especially for brown and American black bear (Can et al. 2014). Understanding how animal species respond to these changes is crucial for both wildlife and forest management, but the number of studies addressing the topic is limited by the need of a long time series of data from different disciplines.

A previous study showed that some bear species can vary their home range based on available mast seeding (Kozakai et al. 2011). However, few studies investigated the spatio‐temporal response of European brown bears to variable seed availability, in particular in low or null mast years. Understanding spatio‐temporal behaviour in this species is key for their effective management and reducing human–wildlife conflicts, which are increasing in Europe (Corradini et al. 2024; Nanni et al. 2024; Sikdokur et al. 2024). Some of these conflicts, especially predation of livestock and damages to crops, can be related to the variation in hard mast production; their number was significantly higher in years with low beechnut production (Bautista et al. 2023).

The Eurasian brown bear ( *Ursus arctos arctos* ), reintroduced in the Italian Alps in the early 2000s from the Dinaric‐Pindos bear population (PACOBACE 2010) to augment the nearly extinct local population, is mainly herbivorous (Frassoni 2001; De Barba et al. 2014). The first dietary analysis based on scats showed that more than 60% of faecal volume (FV) is composed by plant matter, 17% by insects (Hymenoptera) and only 6% by mammals (Frassoni 2001). Among the plant matter, the largest contribution in terms of volume came from the hard mast of Fagaceae and Betulaceae. Frassoni (2001) also reported a seasonal variation in the diet of brown bears in the Alps based on seasonal availability: in autumn, before hyperphagia, beechnuts ( *Fagus sylvatica* 35% FV), hazelnuts ( *Corylus avellana* , 15% FV) and berries from Rosaceae (15% FV) were the main sources of food; in spring, the diet shifted to fresh vegetation and carrions, but still included the remaining beechnuts that account for 15% FV. Oak acorns (*Quercus* spp.) were less consumed in the Alps compared to other regions in Spain (Pérez‐Girón et al. 2022, Cantabrian brown bear) or Italy (Apennine brown bear Ciucci et al. 2014), reflecting the plasticity of the species to exploit different locally available food sources. More recent dietary analysis corroborated the notion of a largely herbivorous diet for the brown bears in the Alps (De Barba et al. 2014), as well as for the Dinaric‐Pindos bear population (Croatia, 80% of the volume based on stomach content, and also Pereira et al. 2021). The abundance of food can also affect the duration of winter hibernation of wild bears, as it occurs in Slovenia where artificial feeding sites provide extra calories throughout the year (Krofel et al. 2017).

In this work we aim to quantify the calories provided by beechnuts and how they are distributed in the study area. This information could explain spatial and temporal patterns of damage to human activities at the landscape level as well as the space use of the Eurasian brown bear at a more local scale. Since beechnuts are the most consumed hard mast by bears in the Italian Alps, we expected that their availability would strongly affect bear spatial behaviour, especially during the hyperphagic phase before denning. In this study, we hypothesise that brown bears would select for beechnut forests more strongly during a mast year than during a poor, or normal year, and would avoid anthropogenic food (i.e., agriculture) to reduce risk of proximity to humans when high‐calorie natural resources are abundant. We also hypothesise that, during mast years, reported damage to human properties (i.e., beehives, livestock, agriculture) would decrease, as bear trophic needs are primarily fulfilled by the large availability of beechnut hard mast. On the contrary, during poor and normal mast years, we expect no difference in selection between beechnut forest and agriculture areas, and we would also expect an overall lower reported damages to human properties.

## 2 Materials and Methods

### 2.1 Study Area

The research was conducted in the Autonomous Province of Trento (hereafter Trentino), Italy (45°.88°–46°.67 N; 10°.99°–11°.78 E), a mountain region of 6200 km^2^ in the Central‐Eastern Alps. This region has a population of approximately 500,000 living from low to mid elevations, with an average of 87 inhabitants/km^2^. In Trentino, forests encompass about 63% of the province; the composition is typically alpine: Norway spruce ( *Picea abies* ) forest represents 32% of the coverage, European beech 14%, European larch ( *Larix decidua* ) 13% and silver fir ( *Abies alba* ) 11%, while the rest is covered by mixed forest of various broadleaves and conifers.

**FIGURE 1:**
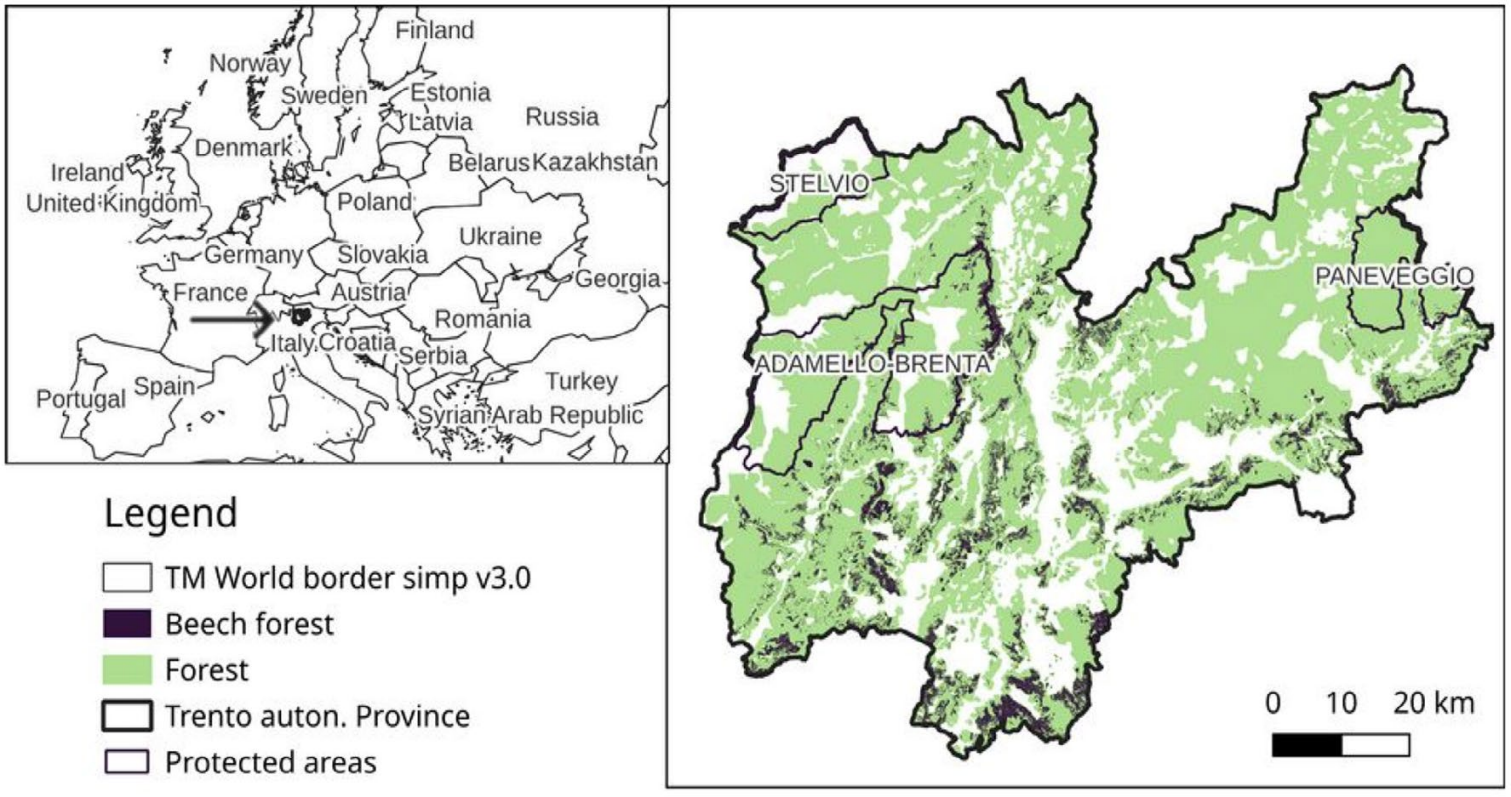
Study area and distribution of forest and beech forest. Left map: Location of the study area ‘Trento Autonomous Province’ in Europe (45°.88°–46°.67 N; 10°.99°–11°.78 E). Right map: Enlargement of the study area, including the three protected areas (Stelvio, Paneveggio and Adamello Brenta Parks) with a focus on forest coverage.

The areas at lower elevations are highly anthropic with intensive agriculture, mainly orchards (i.e., grapes and apples), urban areas and infrastructures. The 10 bears reintroduced from Slovenia in the early 2000s have contributed to build a population of 98 (86–120) individuals in 2023 (Groff et al. 2024); the core area of the population is in western Trentino, mainly in Adamello Brenta Natural Park and its surrounding, while dispersing isolated males were observed occasionally in the eastern part of the province (Figure 1).

### 2.2 Beech Seed Production

Annual seed production by beech and the mast year classification were obtained from multiple sources (Mezzavilla 2015; Hacket‐Pain et al. 2022; Chianucci et al. 2021; Tattoni et al. 2021; see Table 1). The ‘Centro Nazionale Carabinieri per la Biodiversità di Peri’ (CNCBP), a public agency that collects seeds for reproduction in plant nurseries, provided data about the number of seeds and their dry weights from 2007 onwards. The database MASTREE+ (Hacket‐Pain et al. 2022) was queried in order to find data about beech seed production for the years 2007–2021, selecting the nearest sources to our study area. Beech production in the cited sources referred to beech forests managed as high stands. In the study area, there are also forests managed as coppice, a management system that produces fewer beech seeds than high stands (Cutini et al. 2010). In a coppice forest, trees are regularly cut to stumps in order to increase the growth of small stems used for firewood; thus, the trees are smaller and produce fewer seeds. However, from the second half of the past century, coppice management has been progressively abandoned in Italy, in favour of high forest conversion (Coppini and Hermanin 2007). Trentino is no exception, and coppice regards the minority of forests (Nocentini 2009). Therefore, for the purpose of this work, we used the same number of seeds/m^2^ for all types of beech stands. These data allowed us to estimate the annual beech seed production (Mg/ha) in the Northern Alps. For mast level classification, we referred to the quantitative classification proposed by Rohmeder (1972), and adopted also by CNCBP, based on the percent of trees in the stand that produce seeds and on the amount of seeds per tree:

- Level 0, crop failure: trees producing seeds < 10% with any or few seeds per tree.
- Level 1, scarce: 10%–50% trees producing seeds, with few seeds per tree.
- Level 2, good: 50%–80% trees producing seeds, with abundant seeds per tree.
- Level 3, mast: > 80% trees producing seeds, with abundant seeds or overloaded tree.

**TABLE 1:** Beechnut production data used in this work. The values per year (beechnuts/m^2^, weight 1000 seeds (g), calories/m^2^) refer to average measured values for the Alps. The column Source reports ‘This work’ when missing data were averaged over available values for the same mast level across different bibliographical sources. The weight of 1000 beechnuts were provided by Centro Nazionale Carabinieri per la Biodiversità di Peri, Italy, and based on field collection. The amount of calories per square metres was calculated in this work based on the literature cited and experimental measures.

Year	Mast level	Beechnuts/m^2^	Weight 1000 seeds (g)	Calories/m^2^	Source
2007	1	52.0	182.3	39,018	Mezzavilla (2015)
2008	0	40.0	168.35	27,717	Mezzavilla (2015)
2009	3	163.0	190	127,473	Mezzavilla (2015)
2010	2	20.0	168.35	13,859	Mezzavilla (2015)
2011	3	226.8	182.3	170,141	Hacket‐Pain et al. (2022)
2012	0	11.2	168.35	7761	Hacket‐Pain et al. (2022)
2013	3	990.0	198.8	810,078	Hacket‐Pain et al. (2022)
2014	0	0.0	168.35	0	Hacket‐Pain et al. (2022)
2015	0	0.0	168.35	0	Hacket‐Pain et al. (2022)
2016	3	494.9	210.2	428,212	This work
2017	0	10.2	168.35	7096	This work
2018	1	52.0	196.4	42,036	This work
2019	0	10.2	168.35	7068	This work
2020	3	600.0	218.4	539,361	Chianucci et al. (2021)
2021	0	0.0	168.35	0	C.T. (personal observation)

### 2.3 Brown Bear Movement Data

We analysed GPS trajectory data from all available individuals: 16 adult brown bears (12 females and 4 males) using Vectronic GPS–GSM collars (Vectronic Aerospace GmbH, Berlin, Germany) tracked between 2007 and 2021 as part of the monitoring activities undertaken in Trentino according to the interregional management protocol PACOBACE (2010). The capture and handling of brown bears complied with national and international regulations (More details are provided in the Supporting Information and Figure S1). For the study, we considered only the GPS trajectories between September 1st and November 30th, which coincides with the late hyperphagia of bears and the fall fruiting period of European beech. We excluded for each trajectory the GPS locations with missing coordinates or timestamps, those associated with impossible movement parameters (i.e., ‘spikes’) or those in highly uncertain locations (e.g., over a lake), as described in Basile et al. (2014). Because for monitoring reasons high‐frequency bursts occur irregularly, we removed locations to a minimum sampling rate (number of fixes/day) of 1 h (±10 min) using the R package ‘amt’ (Signer et al. 2019). We also discarded trajectories shorter than 14 days and with less than 50 locations and falling outside the study area, this operation did not alter the number of bears monitored. The final dataset consisted of trajectories with irregular sampling rates ranging from 1 to 6 h, and because some individual bears were monitored for more consecutive years, a total of 24 bears‐year.

### 2.4 Damage Data

Data about damages reported to local authorities caused by bears to human activities are collected and managed by the wildlife office of the Autonomous Province of Trento, following the protocols and guidelines from PACOBACE (2010). For each claim, the dataset provided a diverse set of information including a detailed description of the reported damage, location and date, the species of crops or domestic animal involved, the amount of the reimbursement and the individual bear where the identification was possible. For this study, we used all the damages that were certainly attributed to a bear after an assessment by specialised personnel, sometimes corroborated by genetic analysis, collected from 2002 to 2021. Despite the restrictions to people's movement during the 2020 COVID‐19 pandemic, the registration of claims continued normally, because the outdoor work for animal care and agricultural were allowed. People usually report damages to local authorities because of the compensation scheme in place, once verified, all the claims are paid for without monetary limits (PACOBACE 2010). Damages were grouped in the following classes: livestock, agriculture, beekeeping, poultry and other (including various items of human properties such as fences, buildings and garbage bins; see Figure S3) following Corradini et al. (2021). The number of occurrences and amount of reimbursement for each of the above classes were aggregated by month and standardised according to the bear population size for the corresponding year.

### 2.5 Environmental Layers

We developed a set of spatial covariates meant to be informative when modelling bear space use (Peters et al. 2015; Corradini et al. 2024) to test hypotheses regarding habitat selection throughout their monthly range during mast years compared to normal years of beechnut production. Specifically, we mapped the (i) distribution of beech forest based on a forest types vector map (updated after Odasso 2002). We derived (ii) elevation from an airborne laser scanning survey (i.e., LiDAR, with an original spatial resolution of 2 m) and derived the (iii) Terrain Ruggedness Index covariate from it. We derived the (iv) main paved road network from the local infrastructure department (http://sdi‐pat.provincia.tn.it/webgis) and derived the distribution of (v) human settlements, (vi) orchards (i.e., apples and vineyards) and (vii) agricultural fields from the official local cadastral system (http://www.urbanistica.provincia.tn.it/pianificazione/piano_urbanistico_provinciale/cartografia/). We then computed for the paved road network and the human settlement the Euclidean distance of each raster cell to the nearest road/settlement and transformed the resulting proximity maps to exponential decay. For the analysis at the landscape level we also used official road maps (including dirty roads) and a land use map for the Province of Trento, aggregated in seven classes: (1) populated areas, (2) rural areas, (3) pastures, (4) nature reserves, (5) rocks, (6) forests and (7) water (https://webgis.provincia.tn.it/, updated 2022). All land classes are discrete variables, whereas the terrain and distance covariates are continuous (see Table S1 for a detailed description). All the analyses were carried out in WGS 84/UTM zone 32N EPSG:32632 resolution 10 m, and unless stated differently, using QGIS 3.7 (https://www.qgis.org) and GRASS GIS 7.8 (https://grass.osgeo.org/).

### 2.6 Analysis

#### 2.6.1 Calories Estimation

Beechnuts were collected in fall 2020, a mast year, in Val di Sella (Trento, Italy) during the field samples of Chianucci et al. (2021). The seeds were collected in quadrats over a systematic grid in a 0.25 ha plot, following an established protocol (Chianucci et al. 2021). The non‐empty seeds were then oven‐dried at 85°C ± 2°C for not less than 12 h and then reduced to powder with a grinder. The powder was then compressed into 10 pellets of 1 g each, then burned in a Mahler bomb calorimeter. This device measures the increase in temperature of a known amount of water generated from the fuel combustion (beechnuts), allowing to calculate the high heating values (HHV). Benzoic acid and imidazole were used for calibrating the calorimeter before burning the beechnut pellets. The mean caloric value HHV was then multiplied by the annual seed biomass to get food availability maps.

#### 2.6.2 Damage Pattern

Damage pattern was modelled at the landscape level using MaxEnt version 3.4.3 (Phillips et al. 2006, 2022). We used MaxEnt because our data consisted of presence‐only damage locations, for which MaxEnt is particularly well‐suited. Moreover, MaxEnt has been successfully applied in similar human–wildlife conflict studies about bear damages (Khosravi et al. 2023). The locations of damages were used as presence points to identify environmental drivers explaining the different types of damages as in Corradini et al. (2021). We ran MaxEnt using 75% of the points as training and by performing 10 replications with 12,000 background points, using AUC and ROC curves to assess the goodness of the model. An AUC above the threshold of 0.8 is considered as an excellent discrimination (Hosmer et al. 2013). We fitted a linear model to analyse the number of standardised damages over the years using type of damage as an interaction factor to assess the general temporal trend. Differences in monthly amount of damage per bear during mast or poor years were tested with the Wilcoxon test.

#### 2.6.3 Habitat Selection

We fit a second‐order resource selection function to model individual habitat selection during the hyperphagia period as a function of varying beechnut abundance (Johnson 1980). We chose the second‐order (i.e., selection of individual ranges within the population range) because we expect bears, as opportunistic omnivores with non‐territorial behaviour, to have unconstrained movement across their range and to modulate their use of space as a function of sparse food resources. We defined the bear population range as the 100% minimum convex polygon (MCP) of all 95% individual MCPs derived from the *hr_mcp* function in the R package amt (Signer et al. 2019). We estimated monthly ranges for each bear (from September to November) based on the 95% utilisation distribution derived from the Biased Random Bridges using the BRB function in the R package ‘adehabitatHR’ (Calenge 2006). We set the drift parameter (‘Tmax’) to 21,600 s (or 6 h) based on the longest sampling rate between GPS locations, with any steps exceeding this interval being excluded from the calculation. We set the minimum distance between successive relocations (‘Lmin’) to 100 m, thus accounting for GPS errors in intensive use areas or resting, and the minimum smoothing parameter (‘hmin’) to 500 m to account for uncertainty of the habitat map. We applied a used‐available design (Manly et al. 2002) by randomly drawing 500 locations within each monthly range as ‘used’ and ‘available’ random sites in a 1:5 used/available ratio across the population range, removing only locations associated with no values in the environmental layers. This resulted in a total of 24,500 used and 121,242 available locations.

We tested our hypotheses by fitting an ecologically informed generalised linear mixed model (GLMM) using Template Model Builder (TMB) with a binomial error distribution. Specifically, our fixed effects tested for differences in habitat selection between mast and non‐mast years (boolean) for three key habitat types: beech forest, orchards and agriculture fields. The model included additional fixed effects to control for environmental variability (elevation, as both linear and quadratic effect to account for potential non‐linear relationships and terrain ruggedness) and anthropogenic disturbance (distance from main paved roads and human settlements). Before fitting the model, we tested for collinearity (|*r*| ≤ 0.6) among all covariates and scaled all numeric predictors to a mean of zero and a standard deviation of one. We set a weight (*W*) of 1 for each used location and 1000 for each available location (Muff et al. 2020). As a random effect, we included individual bear ID as random intercepts to account for autocorrelation (Gillies et al. 2006). After model fitting, we evaluated model performance using functions in the R package ‘performance’ (Lüdecke et al. 2021).

## 3 Results

### 3.1 Beech Mast Time Series

The experimental burning of nine pellets (one pellet was discarded because of incomplete burning) produced an estimation of the caloric content of beechnuts equalling 4116 cal/g ±437. This result is comparable with the calorie content reported in literature for the American beech ( *Fagus grandifolia* ) 5000–6000 cal/g; however; the literature did not report the method used for calories estimation (Jaffa 1908; Rosengarten 2004). Even if nut size can vary based on environmental conditions, beechnuts from American beech are generally larger than those of European beech; we think that our estimates can be considered realistic (*source:* https://plants.ces.ncsu.edu/plants/fagus‐grandifolia/common‐name/beechnut‐tree/).

Table 1 summarises the results of the research about mast production across sources and the calories count, limited to the years when CNCBP collected and weighed the beechnuts (2007–2021). Mast years, level 3 of the Rohmeder's scale, were: 2009, 2011, 2013, 2016 and 2020; only the year 2010 had a mast level of 2. However, in 2010 the number of available nuts per square metre was only 20, a value closer to a poor year for our study area. In fact, the amount of beechnuts/m^3^ and calories/m^2^ was not significantly different among mast level 0, 1 and 2 (Wilcoxon test, *p* > 0.05) but it was among levels 0 and 3 (Wilcoxon test *p* < 0.005). Thus in the following analysis we grouped mast levels 0, 1 and 2 into a single class (non‐mast year) and considered only when mast level was 3 as mast years.

### 3.2 Bear Habitat Selection

**FIGURE 2:**
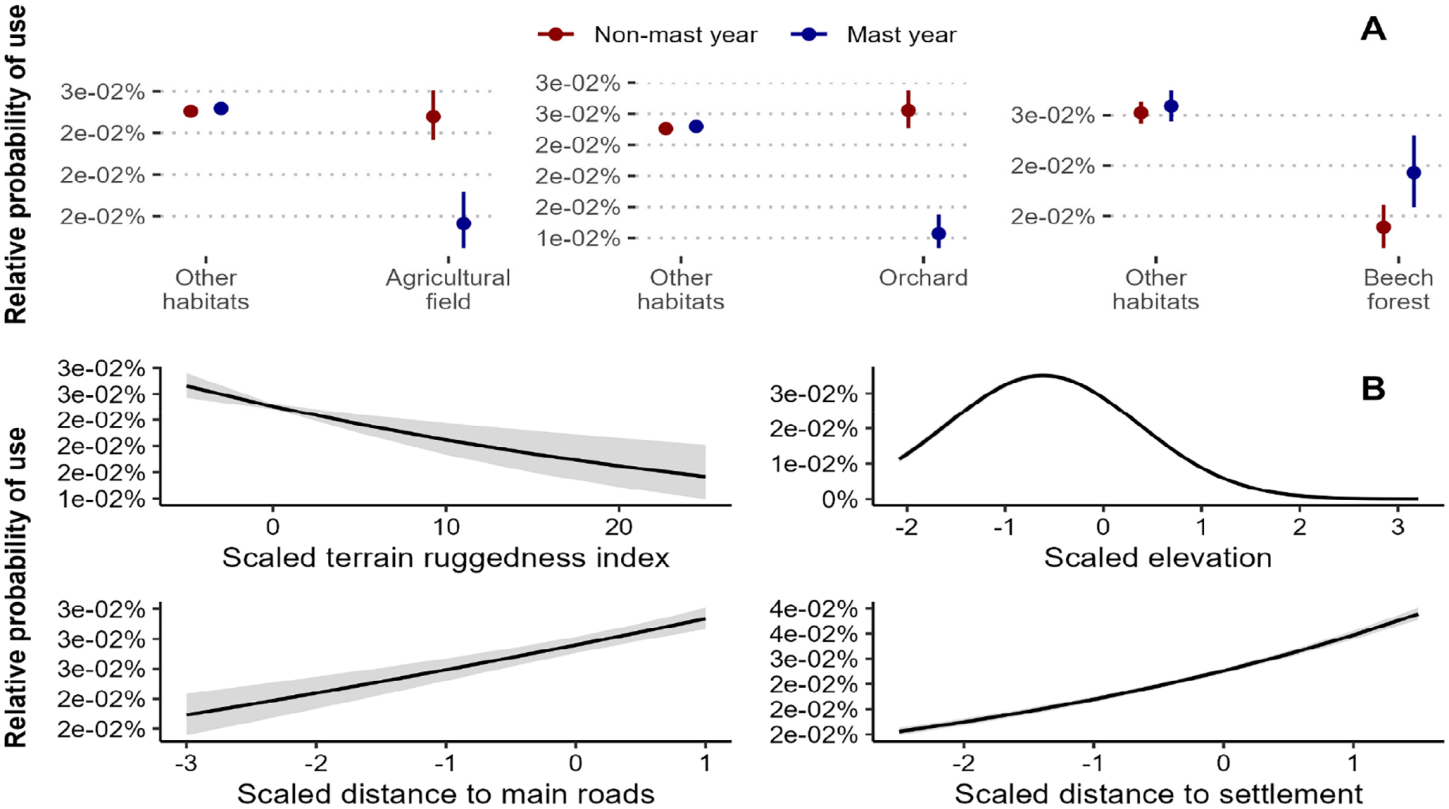
Fitted regression coefficients and 95% confidence bands, estimated via glmmTMB, for the empirical relationship between monthly range selection and (A) natural (beech forest) and anthropogenic habitats (orchard, agriculture field) between mast and non‐mast year, and (B) terrain ruggedness, elevation, distance to main roads and distance to human settlement. Predicted probabilities of use (*y*‐axes) were calculated using the ggeffects R package (Lüdecke 2018). In panel A, ‘True’ indicates mast years (high beech seed production) and ‘False’ indicates non‐mast years (low seed production). True and False refer to Mast year (True: mast year, False: non‐mast year for beech seeds production).

We derived a total of 40 individual monthly ranges during non‐mast years (mean = 48.93 km^2^, SD = 29.56 km^2^) and 9 monthly ranges during mast years (mean = 63.30 km^2^, SD = 18.64 km^2^). When selecting for monthly ranges across the population range, we observed that bears selected for beech forests (*b* = +0.104, *p* < 0.05) significantly more during mast years than during non‐mast years, and that similarly avoided orchards (*b* = −1.057, *p* < 0.001) and agriculture fields (*b* = −0.658, *p* < 0.001) in mast years (Figure 2A), supporting our hypothesis. Bears avoided proximity to human settlements (*b* = +0.229, *p* < 0.001) and to main paved roads (*b* = +0.062, *p* < 0.001), and selected for lower elevation (linear effect: *b* = −0.651, *p* < 0.001), but avoided extreme values (quadratic effect: *b* = −0.529, *p* < 0.001) (Figure 2B), as expected. Interestingly, we observed that bears did not select for terrain ruggedness during the study period (*b* = −0.026, *p* < 0.001; September 1st and November 30th) (Figure 2B), contrary to previous findings (Corradini et al. 2024). Model performance assessment evidenced that the variance inflation factor for the full model was less than 2 for all covariates, thereby indicating low correlation, and the conditional *R* ^2^ was 0.193.

Only five individuals were monitored during both poor and mast years, and thus comparisons at home range level were performed, but cannot be generalised (Figures S1, S5 and S6).

### 3.3 Temporal Pattern of Damages

The total number of damages in the database was 2997, with a steady increase over time following the trend of the bear population (Figure S2).

The most reported claim in nearly 20 years involved beekeeping activities (1005), followed by predation on livestock (719, of which more than half were sheep and goats) various damages to agricultural activities (603, especially eating fruits), poultry (331, of which 27 rabbits) and miscellaneous damages to objects and private property (339). The average number of claims per year, standardised according to the number of bears, was 3.9 (SD = 2.2); in detail: agriculture 0.67 (SD = 0.29); beekeeping 1.4 (SD = 0.8); livestock 1.03 (SD = 0.77); other 0.31 (SD = 0.51); poultry 0.46 (SD = 0.16). Annual values were calculated for comparison with literature and to fit linear models.

In the database, the genotype of the bear responsible for the damage was only reported in about 17% of the cases (488 out of over 2900, by 61 individual bears). Nevertheless, we observed that within the data that reported the genetics, three single bears (two males and one female) were responsible for over 30 claims each, while the majority of identified bears (46) for less than 10.

The average amount of reimbursement was 513 Euros with a large variability (median 260, min 125, max 13,525 Euros). Yearly compensations were on average 72,405 euros per year (min 8700 euros in 2003—maximum 150,748 in 2019).

No significant difference was found in the costs of damages claimed during mast and non‐mast years; this could be explained because of the high variability in the value of the single event (Wilcoxon test *p* > 0.01, Figure S4).

Overall, the various kinds of damages followed a seasonal pattern according to the availability and location of the anthropic food source. Livestock predation had a peak around August (average 7.6, SD = 2.3) and it was also high from late spring, when the animals are taken outside and at higher elevation for pasture, and decreases after September. Similarly, the depredation of crops and fruits occurred mostly from June to October (peak in September, average 7.8, SD = 2.5) when they were ripe. Beehives were more damaged by bears in April (average 12.7 complaints per month, SD = 3), probably because during springtime the hives are kept at mid elevations and later during the summer moved above the tree line. The other classes did not show a great monthly variability (Other mean 3.0, SD = 1.13, Poultry mean 2.9, SD = 1.25), showing similar temporal trends in mast and non‐mast years. During the months of hibernation (November to March in the study area), we observed a general drop in any type of reported claim.

**FIGURE 3:**
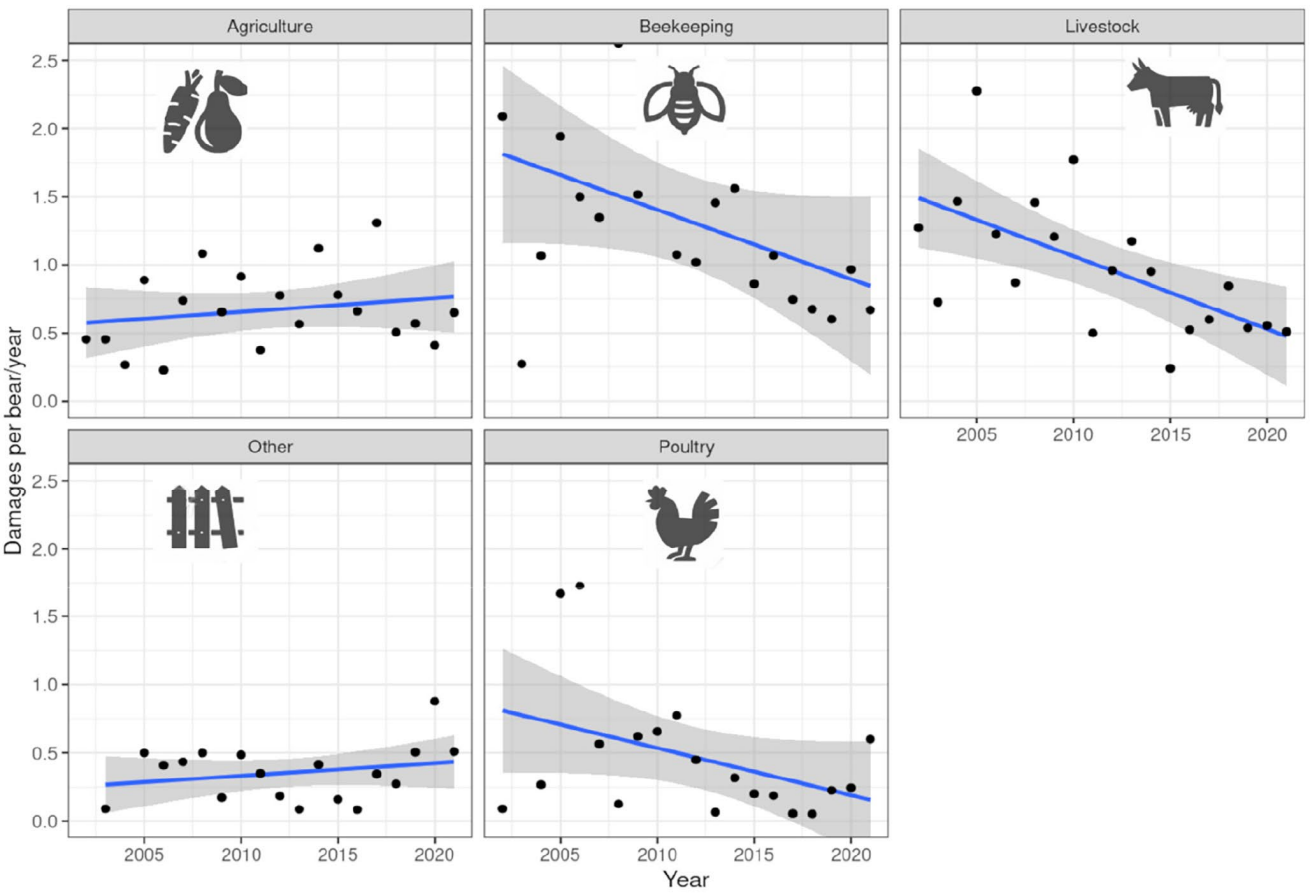
Trends of the yearly number of damages per type of damage, standardised by brown bear population size in the Italian Alps (2002–2021), shaded areas represent 95% confidence intervals.

The annual trend of claims per bear between 2002 and 2021 varied according to the human activity considered. The standardised damages to beehives, livestock and poultry decreased over time, with linear regression slopes (±standard errors) of −0.051 ± 0.031, −0.053 ± 0.031, and −0.35 ± 0.032, respectively. In contrast, the damages to agriculture and miscellaneous categories exhibited uncertain trends, with slightly positive slope estimates (0.010 ± 0.018 and 0.009 ± 0.031), but large standard errors (Figure 3).

**FIGURE 4:**
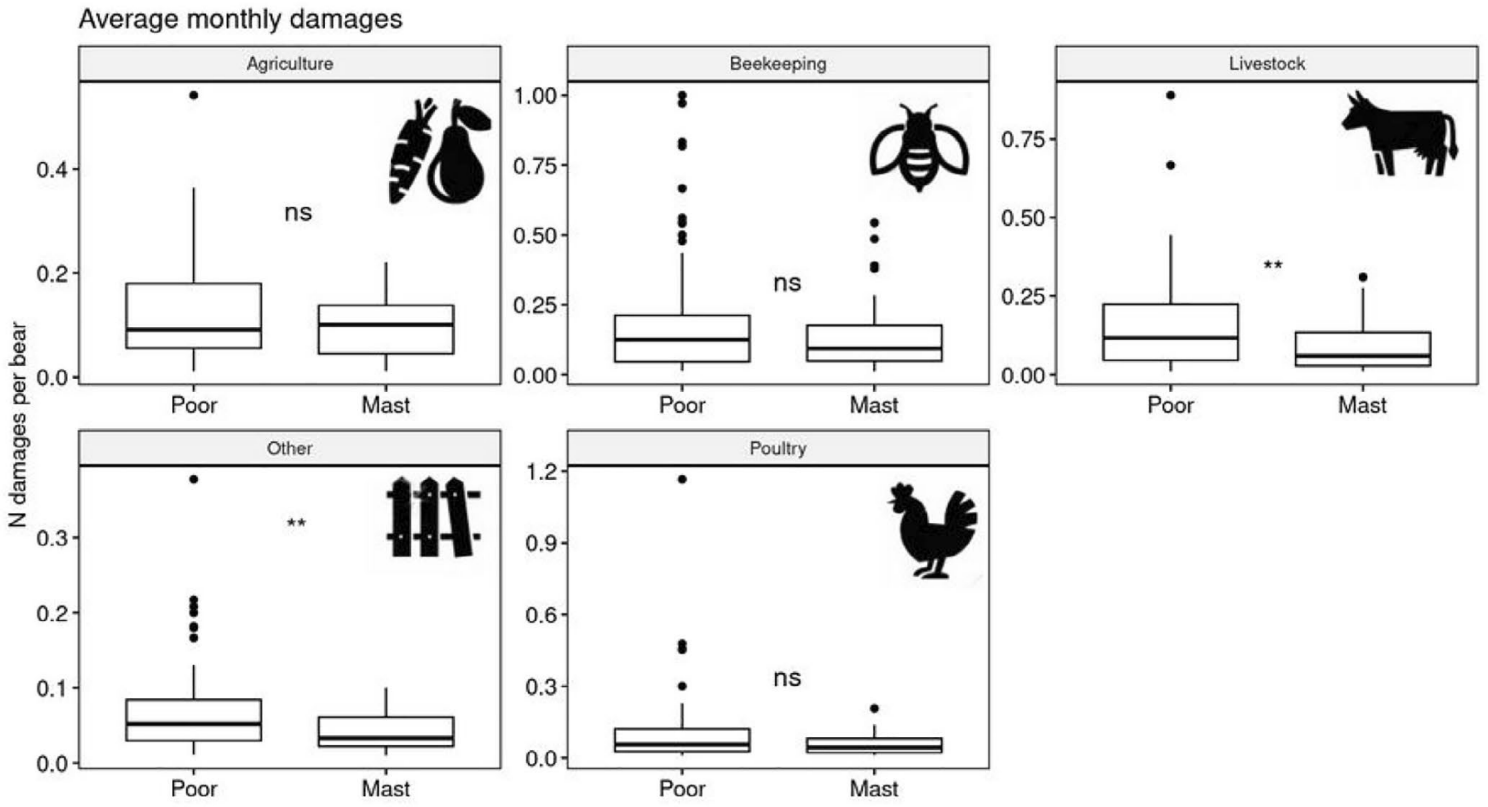
Average monthly damages per brown bear according to damage type in relation to beech mast year.

The average number of damages per bear was lower in mast year, but the difference was significant only for livestock and other categories (*p* < 0.01, Wilcoxon test) and not significant for beekeeping, agriculture and poultry (*p* > 0.01, Wilcoxon test). During poor years, we can expect an increase in livestock attacks by bears of approximately 66.67% (Figure 4).

### 3.4 Damage Pattern at the Landscape Level

**FIGURE 5:**
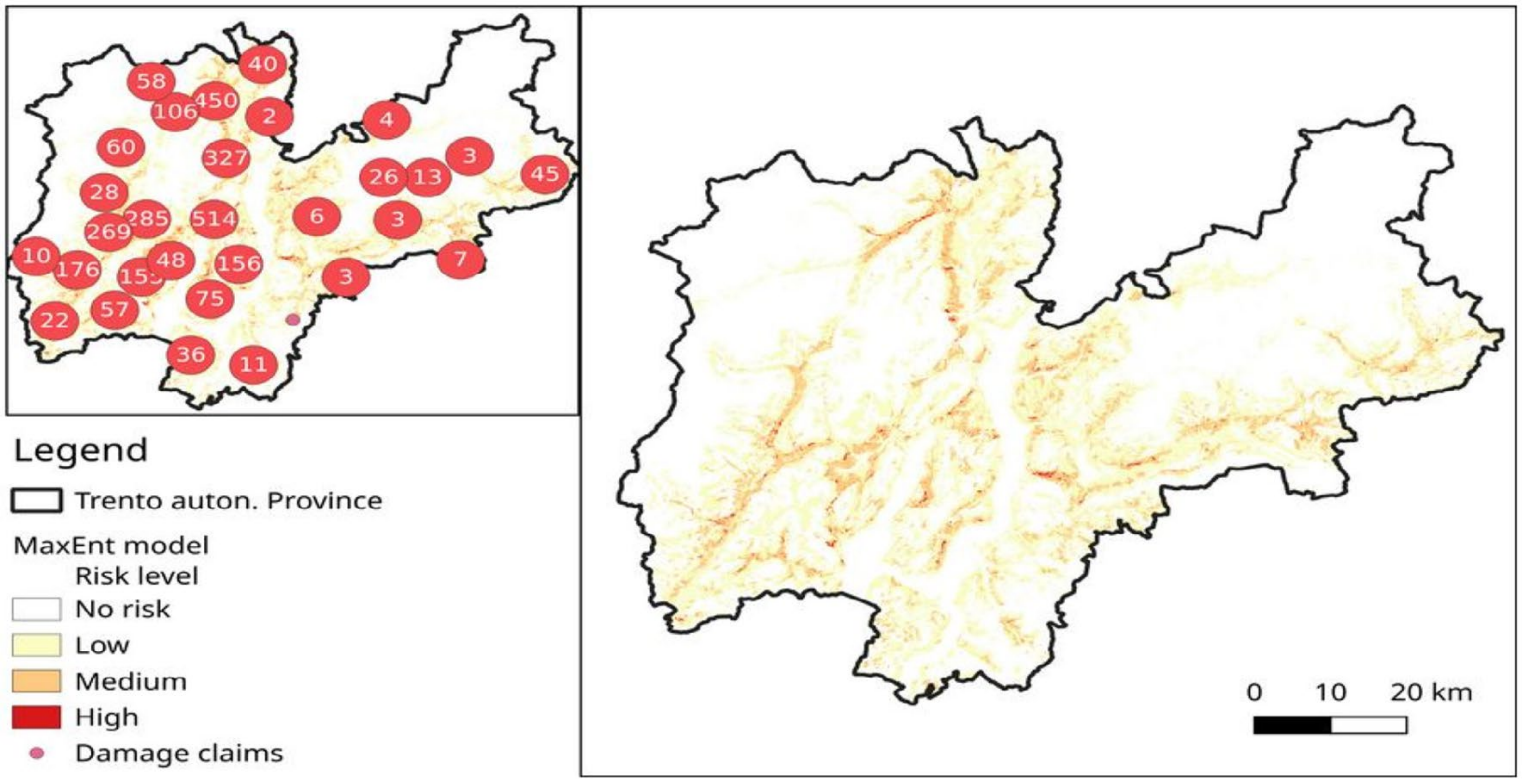
Large map: The potential risk of damage by brown bear in Trentino, Central‐Eastern Alps, reclassified from MAxEnt modelling. Top left inlet: Number of damages claimed from 2002 to 2021, aggregated in a radius of 10 km. Risk levels: No risk: < 0.25, low: 0.25–0.50, medium: 0.5–0.75 and high: 0.75–1.

The pattern of damages in the whole study area was modelled in MaxEnt; the results of 10 split‐sample models were: average AUC was 0.822, SD: 0.006 (model output details are reported in Figures S7 and S8). The probabilistic output map was reclassified as follows: 0–0.25: no risk; 0.25–0.5: low risk; 0.5–0.75: medium risk; 0.75–1: high risk of bear damage and is reported in Figure 5.

The areas of land use most at risk were agriculture and pasture areas, close to beech forests and distant from large population centres and infrastructures (Table 2). The altitudinal range more at risk was between 750 and 1400 m in moderate slopes (< 20%), and the probability of damage decreased rapidly from 2 km distance from the edge of the beech forests and from 3 km from forest roads. The extent of the land classified at medium and high risk was respectively 422.1 and 15.9 km^2^, corresponding to 6.8% and 0.25% of the province.

**TABLE 2:** MaxEnt modelling results: variable contributions explaining the probability of occurrence of a damage by the brown bear in the Italian Alps.

Variables	Percent contribution	Permutation importance
Land use	42.5	15.3
Distance from beech forest	28	36.3
Slope	10	23.1
Elevation	8.9	18.4
Distance from forestry roads	5.1	2.2
Aspect	3.2	4.2
Distance from roads (paved)	2.4	0.5

## 4 Discussion

Human–wildlife conflict is a significant issue for large carnivore conservation, with damage to property being one of the primary types of negative interactions. Understanding the patterns of such damage at a local level and anticipating risks in both time and space can mitigate these conflicts and provide insights for adaptive management.

Here, we analysed the bear‐beech ecological relationship using over two decades of bear damage claims and beech mast historical records at the landscape and bear home range level. We also investigated the temporal pattern of such damage at a monthly resolution. European level studies (Bautista et al. 2017, 2019) usually reported the general pattern of bear damages and compensation on an yearly basis. In the Trentino region, the current annual damage ratio was the same order of magnitude as previously estimated by Bautista et al. (2017), but lower in value. The annual ratio of claims decreased from 4.4 (SD 1.8) in 2017 to 3.9 (SD 2.23) in this study, since we used a longer time series, and the value was updated to the observed decreasing trend in damages per bear.

We demonstrated that the annual variation in beechnuts had an influence on bear behaviour, which in turn influences the pattern of annual damages to human activities, particularly livestock, which were more affected during non‐mast years. The reconstruction of beech productivity in the study area, with mast years in 2013, 2016 and 2020 matched mast years reported at a broader scale (Chianucci et al. 2021), another evidence of the large spatial synchrony of masting in beech trees.

Despite the increasing number of bears and damages over time, we observed a reduction in the ratio of claims to livestock, small animals and bee hives, reinforcing the idea that implementing prevention measures is an effective tool to reduce such negative interactions. In the study area, about 200 prevention measures per year have been operational since 2018, including electric fences and dogs (Groff et al. 2024). Damages to agriculture and miscellaneous property showed an uncertain trend. We speculate that large fields and orchards, which in the study area are often bordering large patches of forest, are more difficult to protect with electric fences compared to livestock or beehives. By definition, miscellaneous damages accounted for a very diverse set of objects that are probably not the main target of bear attention but something that bears found on their way to a food source. In order to find a trend, this class should be further divided and investigated; however; damages to human properties were the class with the lowest number of records, so the trend may be masked by other factors as in other areas (Sikdokur et al. 2024). The number of claims per bear in Europe is generally independent from the population size (Bautista et al. 2017) and it is more affected by management and land cover (Treves et al. 2011). The importance of land cover is confirmed in our study area, both at landscape and individual level. In fact, all the types of damages showed a decrease, except for agricultural claims, which showed an uncertain pattern. According to the model at landscape level, medium and high‐risk areas are primarily concentrated in agriculture and pasture lands, situated far from large population centres.

The pattern of damages fits well also with the results of bear movement analysis. The animals used beech forest regardless of the abundance of seeds, but moved more into agricultural land when the beech crop was poor. The location of intensive agricultural areas close to the forest edge creates the opportunity for bears to feed on apples, grapes and corn without moving far from beech forests. While the GPS tracking data provided high‐quality spatial coverage during the key period of beech masting, there is a limit in the interpretation due to the sex bias, as most GPS‐tracked individuals were female, whereas the majority of identified damage cases involved males. This mismatch limits our understanding of potential sex‐specific patterns in damage behaviour. This gap can be addressed in the future if more male bears will be tracked and, generally, it is necessary that individuals are monitored for a longer periods including at least one mast year. Another limit of this study issues from the damage database, where only a subset of damage events were subject to genetic analysis for individual identification. As a result, only a minority damage cases can be linked to a certain bear. This dual bias limit the assessment of individual and/or sex‐specific patterns of human‐bear conflict and we encourage the collection of DNA in all possible cases.

We found a significant relationship between bear home range size and beechnuts production and caloric contents only for four bears, but not for all the population (see Figures S5 and S6). This is probably due to two reasons: the great individual variability in home range size of bears (Peters et al. 2015) and the scattered data availability across years, because only five individuals were monitored during both poor and mast years (Figure S1). A result based on such a small sample should be interpreted with great care and used as a hint for further investigation to understand if these results can be generalised for the population. This underlines once more the importance of long‐term monitoring for ecological research that can provide useful outcomes for management and conservation.

In this study, we also aimed to disentangle the temporal pattern according to the type of damage and abundance of beech seeds. Damage to apiaries and agriculture happened more during spring and summer, and this could also explain why the relationship with beechnut abundance was not significant. The abundance of other food sources in other times of the year, especially fleshy fruits (Tattoni, Chianucci, et al. 2019; Tattoni, Soardi, et al. 2019; Pérez‐Girón et al. 2024) could be responsible for inter annual variation of damages. Conversely, livestock predation peaked in late summer and autumn, in coincidence with the availability of beech mast, and we observed a significant reduction in such claims. Most of the reimbursement belonging to the category ‘others’ regarded fences, likely ruined during attacks on livestock. Damage to beekeeping occurred when beechnuts were not available and that could explain why there was no difference in mast and not mast years. During mast years, the monthly average of damages per bear to livestock was lower than in poor years. Damage to livestock occurred in summer and autumn, when the availability of natural food decreases the level of predation. Under a management perspective, it is important to focus on the peculiarities of a given bear population, since resource use varies according to location. Managers can intensify the information campaign about the free distribution of preventive measures in some areas during spring or contact beekeepers during winter in order to be operative in the correct time. In the Apennines for example, the peak of all kinds of damages were observed in summer and fall, with livestock depredation and apiaries prevailing over other types of damages in terms of claims (Galluzzi et al. 2021).

When analysing the patterns of claims, the issue of the so‐called, ‘problem bears’, that is individuals that cause significantly more damage compared to others, should not be overlooked. This likely holds true also in this study area, even though caution must be used due to the limited numbers of claims that could be linked to an individual bear. In addition, certain bears can specialise in targeting certain anthropogenic resources such as apiaries (Krofel et al. 2020) or equine (C. Groff, personal observation), thus interfering with the temporal and spatial occurrence of reimbursement requests. Because of the individual variability, the pattern of damage can vary even within the same bear population over time, as noted by Zarzo‐Arias et al. (2020).

In conclusion, the abundance of natural food (beechnuts) in a specific time of the year affected bears' attraction to anthropogenic food. Even in a different management scenario, where bears are hunted and provided with additional food, Pereira et al. (2021) showed that bear usage of supplemental feeding increased during years of beech crop failure.

Climate change also plays a role in this dynamic: increasing temperatures may lead to more frequent mast years (Bogdziewicz 2021), followed by a crop failure, with unknown effects on the extent of damages (Bautista et al. 2023).

The occurrence of a mast year for beech can be anticipated by bioclimatic models, given the well‐known relationship between masting and previous summer temperature (Bajocco et al. 2021; Chiavetta and Marzini 2021; Journé et al. 2023) and confirmed by field observations of the flowering tree during spring, allowing managers to know in advance the (un)availability of this important food resource. Managers could devise in advance various strategies to reduce bear damages in poor years, including targeting specific areas with prevention measures, moving cattle in a different areas, or implementing temporary diversionary feeding sites to keep bears in the forest (Garshelis et al. 2017).

The location of areas of potential risk can also be useful to managers in planning the distribution of prevention measures in the eastern part of the region, where bear presence is still occasional, thus reducing losses and containing human‐bear conflicts.

## 5 Conclusions

This longitudinal study describes the spatial and temporal patterns of beech masting and bear‐related damages over a 20‐year time of observation. We observed a reduced amount of damages to livestock and property during mast years, as reported in the literature by Bautista et al. (2023) and Pereira et al. (2021). During years of crop failure, bears use more agricultural and pasture lands near beech forest, and the number of damages to livestock increases. For example, in anticipated poor mast years, prevention measures should be prioritised in high‐risk agricultural and pasture areas, livestock movements can be adjusted and supplemental feeding may be temporarily considered to keep bears within forested areas and reduce forays into human‐dominated landscapes. The damage risk map at the landscape level and the observation of beech productivity can support the above adaptive management strategies.

## Author Contributions

**Clara Tattoni:** conceptualization (lead), formal analysis (equal), investigation (lead), methodology (lead), software (equal), visualization (equal), writing – original draft (lead), writing – review and editing (lead). **Andrea Corradini:** conceptualization (equal), formal analysis (equal), methodology (equal), writing – original draft (equal), writing – review and editing (equal). **Francesco Chianucci:** formal analysis (supporting), methodology (equal), writing – review and editing (equal). **Marco Ciolli:** conceptualization (equal), investigation (equal), methodology (equal), supervision (equal), writing – review and editing (equal). **Roberta Giusti:** formal analysis (supporting), software (equal). **Natalia Bragalanti:** data curation (lead). **Francesca Cagnacci:** supervision (equal), writing – review and editing (supporting). **Adriano Martinoli:** funding acquisition (equal), supervision (equal), writing – review and editing (equal). **Damiano G. Preatoni:** supervision (lead), writing – review and editing (equal). **Francesco Bisi:** investigation (equal), methodology (equal), writing – original draft (equal), writing – review and editing (equal).

## Ethics Statement

We used GPS data from collared bears that were collected under Directorial Decree Protocol 0015137 PNM of 30 July 2015. We do not have permission to share bear damage data set, they must be asked to the owner, Province of Trento.

## Conflicts of Interest

The authors declare no conflicts of interest.

## Supporting information


Appendix S1

## Data Availability

We used public available maps (cited sources). Bear damage data set is available from Ufficio foreste e fauna Provincia Autonoma di Trento. Scripts are stored in the git‐lab repository https://gitlab.com/clara.tattoni/pasciona.
