# Supplementary material for: Behaviour of Brown Bears Under Fluctuating Resource Availability
Source: Ecol Evol. 2025 Jun 27;15(7):e71693. doi: 10.1002/ece3.71693 (PMC12204723; doi:10.1002/ece3.71693)
Supplement: Supplementary file 1 — Appendix S1 [file ECE3-15-e71693-s001.pdf]

Supporting information for

Tattoni Clara *a; Corradini Andrea b; Chianucci Francesco c; Ciolli Marco d,e; Giusti Roberta e; Natalia Bragalanti f; Francesca Cagnacci b; Martinoli Adriano a; Preatoni Damiano G. a; Bisi Francesco a

Behaviour of Brown Bears Under Fluctuating Resource Availability

a Dipartimento di Scienze Teoriche e Applicate, Università degli Studi dell'Insubria, Varese, Italy
b Fondazione Edmund Mach, San Michele all'Adige (TN), Italy
c CREA, Research Centre for Forestry and Wood, Arezzo, Italy
d DICAM - Università di Trento, Italy
e C3A - Centro Agricoltura Alimenti Ambiente, San Michele all'Adige (TN), Italy
f Ufficio foreste e fauna Provincia Autonoma di Trento, Italy

* Corresponfing author: Clara Tattoni, Dipartimento di Scienze Teoriche e Applicate, Università degli Studi dell'Insubria, Varese, Italy clara.tattoni@uninsubria.it

doi: 10.1002/ece3.71693

Handling of brown bears

In Italy, the brown bear is currently protected under European (Habitats Directive 92/43/EEC, incorporated in the Italian legislation via D.P.R. 357/97) and Italian Laws (L. 157/92), and included as a strictly protected species in the Bern Convention (1979). Under derogation of the aforementioned Habitat Directive, approved by the Italian Institute for Environmental Protection and Research (ISPRA), it is permitted to capture animals for research, public safety, and damage prevention purposes. Within these limits, all trapping and handling operations of wild brown bears are regulated by the “Interregional action plan for the conservation of the brown bear in the central-eastern Alps” (PACOBACE, 2010), approved by Directorial Decree n. 1810 of 5 November 2008 and amended by Directorial Decree Protocol 0015137 PNM of 30 July 2015.

Figure S1 Overview of the data availability of radio-tracked bear during the study period: monitored timed in blue and mast years, red vertical lines

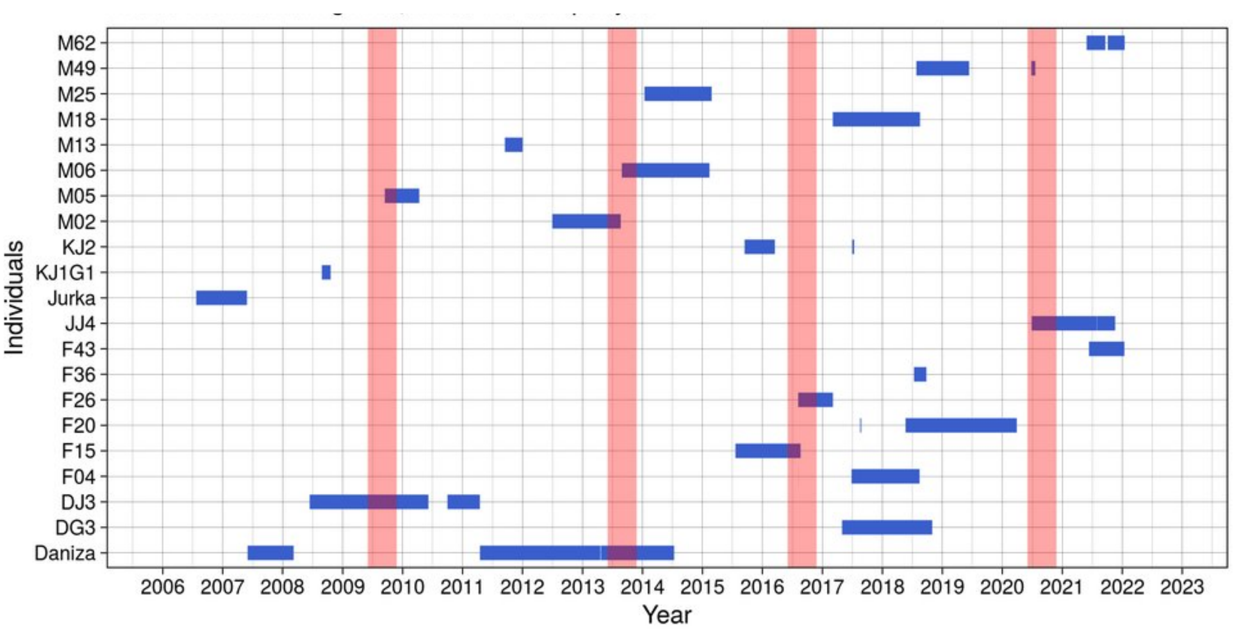

Figure S2 Trends of bear population, number of damages and compensation paid by the public administration in the Italian Alps (2002-2021).

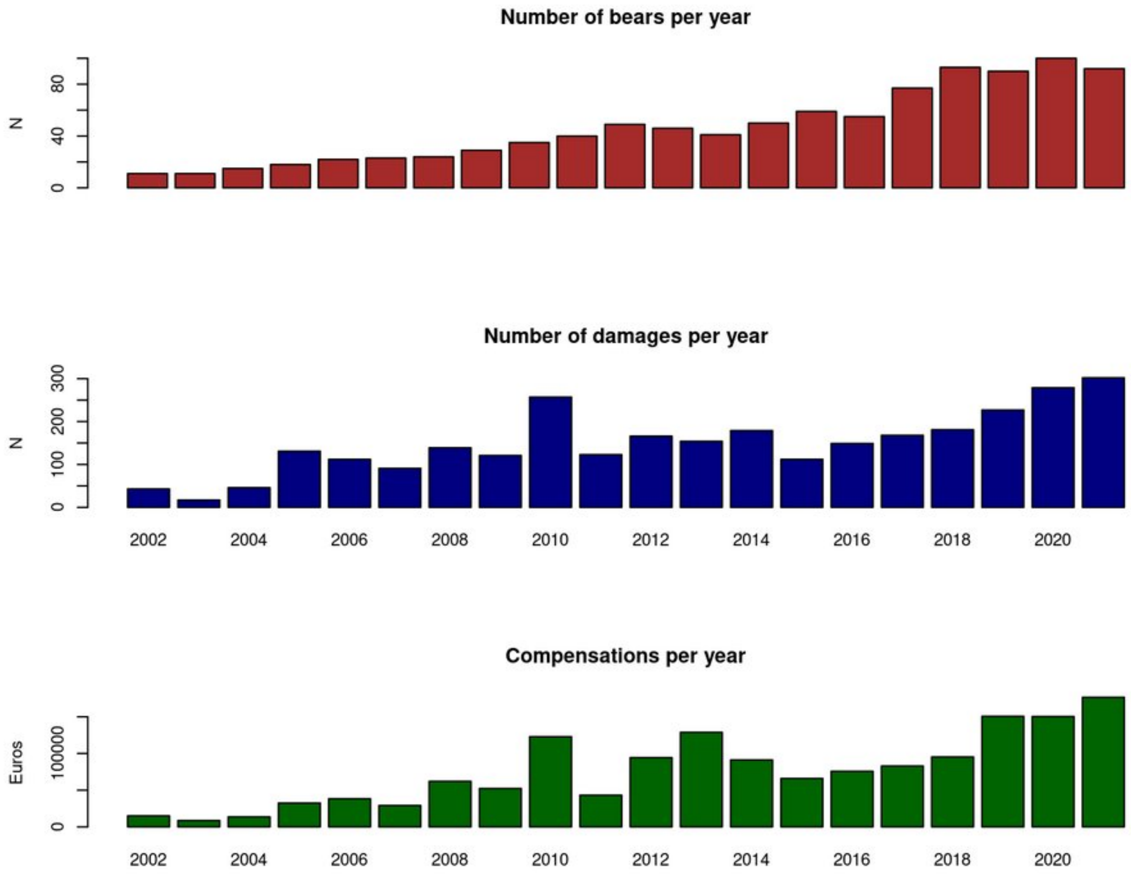

Figure S3 Total number of bear related claims reported to the public administration in Trentino (Italian Alps , 2002-2021).

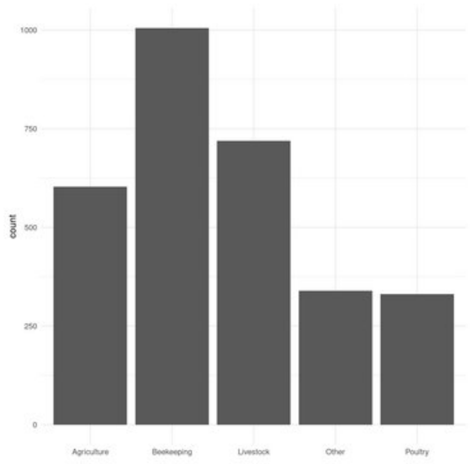

Figure S4 Trends of reimbursements paid yearly by the public administration to compensate for bear-related damages in Trentino (2002-2021). Euros are standardided to bear population size for the given year.

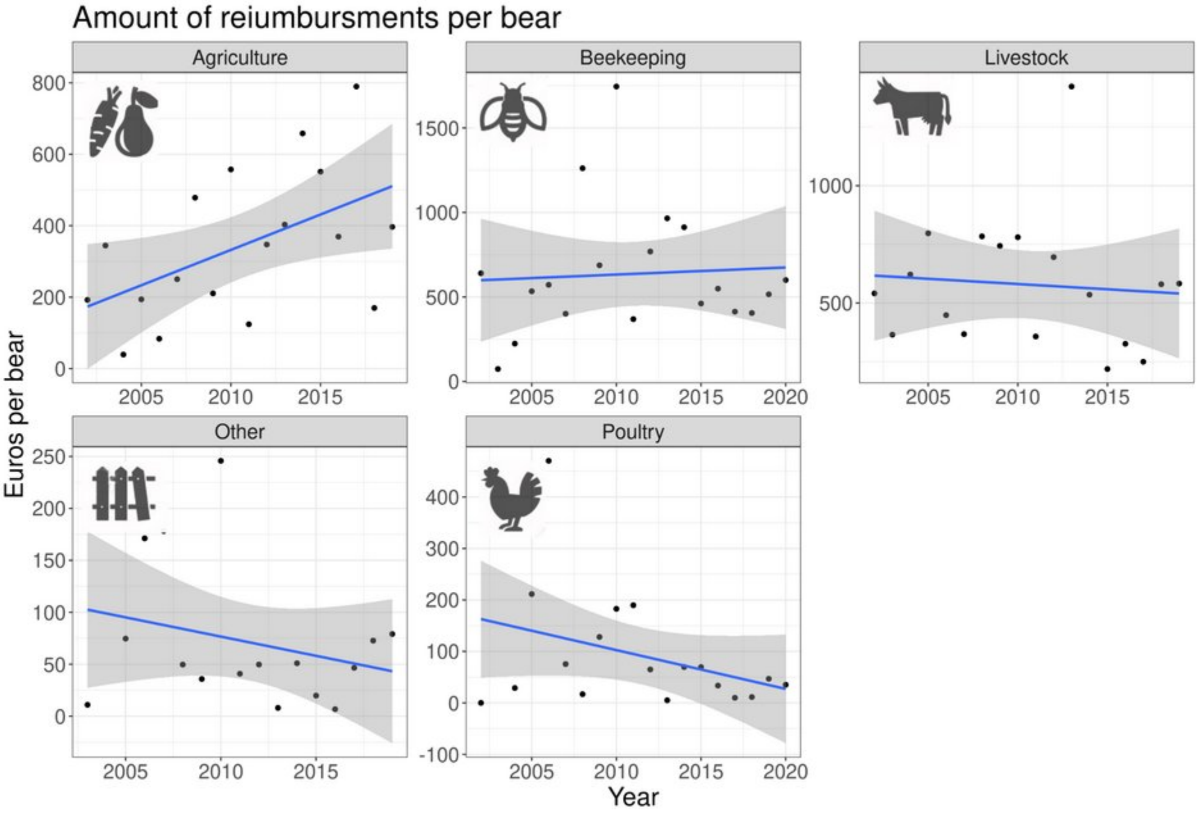

Figure S5 Difference in monthly HR size (95% UD estimated using Biased Random Bridges BRB) for the five individual bears monitored in both beech mast and poor years in the Italian Alps. Despite HR size showing a great individual variability, the bears used a smaller HR during mast year, except the female JJ4.

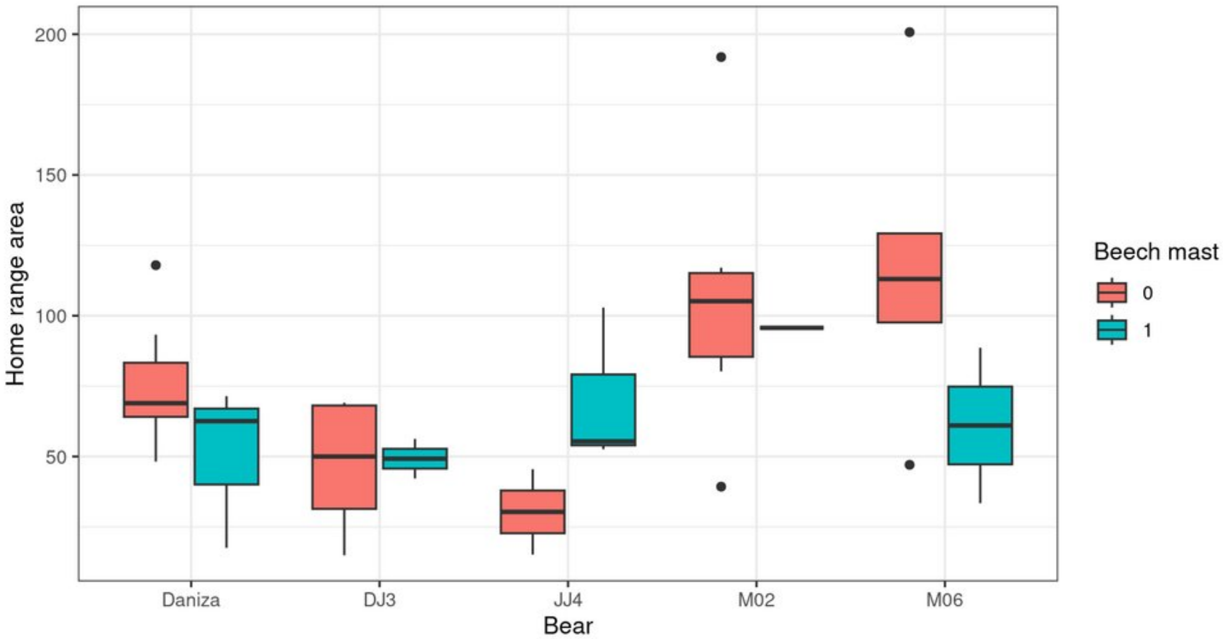

Figure S6 Relationship between HR (95% UD estimated using Biased Random Bridges) and calories availability for the five bears monitored during both mast and poor years.
HR size decreases when beechnuts are more abundant and provide more energy for 4 bears out of five. Kcal pixel was significant (P <0.05 in linear model)

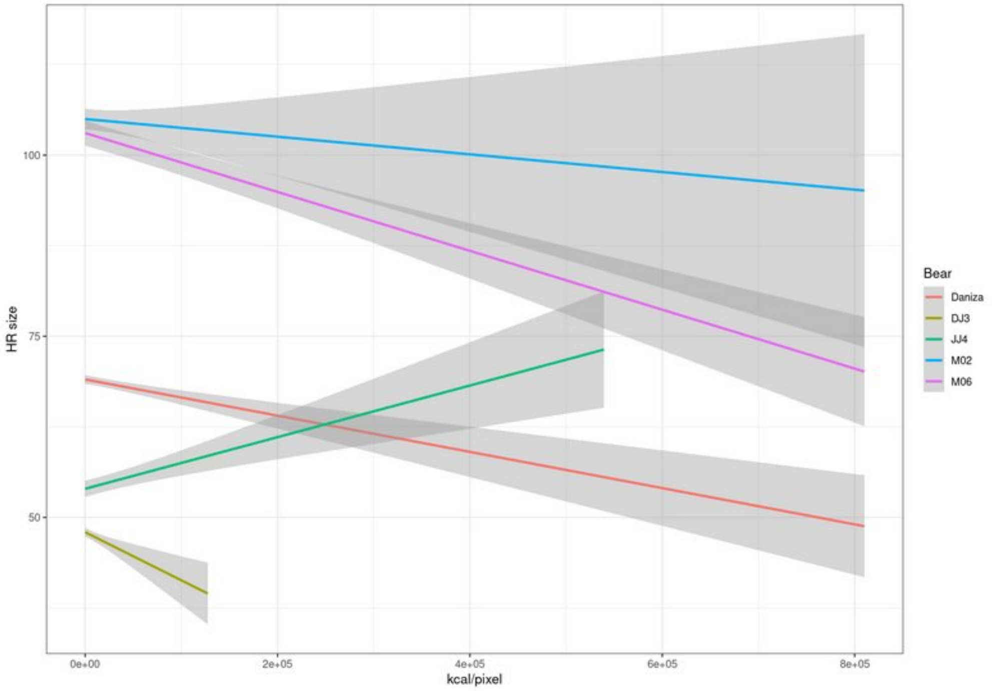

Figure S7 Results from MaxEnt modelling. Response curves displaying how each environmental variable affects the probability of occurrence of a damage provoked by an Eurasian brown bear in the Italian Alps. The curves show the mean response of the 10 replicate Maxent runs with error bars (for land use) and standard error in grey for the other variables (very small and not always visible). Red horizontal dashed line marks 50% predicted probability.

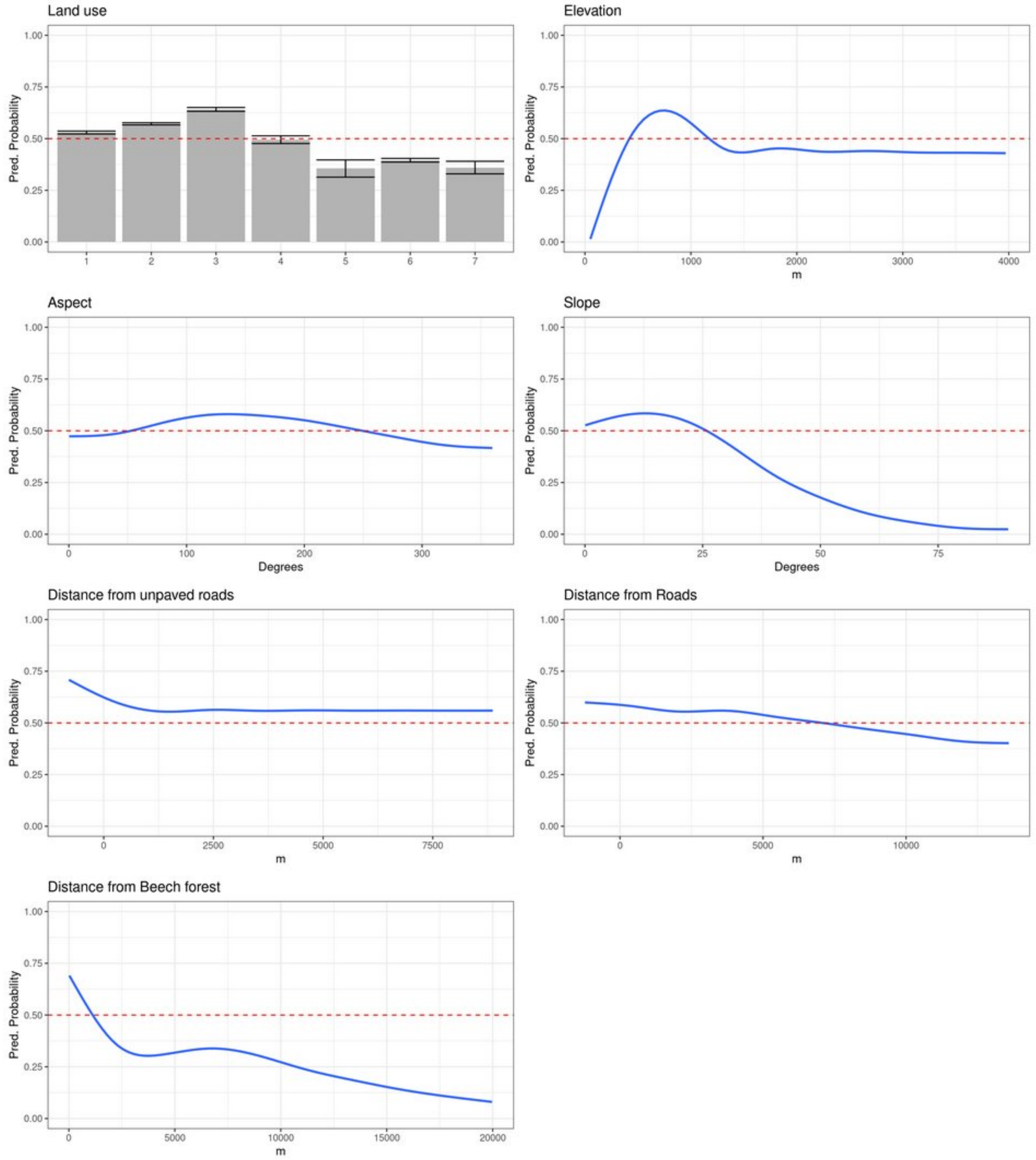

Figure S8 Results of jackknife test of variable importance for MaxEnt model about the probability of occurrence of brown bear related damage, averaged over 10 runs. Elevation is the environmental variable that contributed most to AUC, whereas distance from beech forest variable has the most information that isn't present in the other variables.

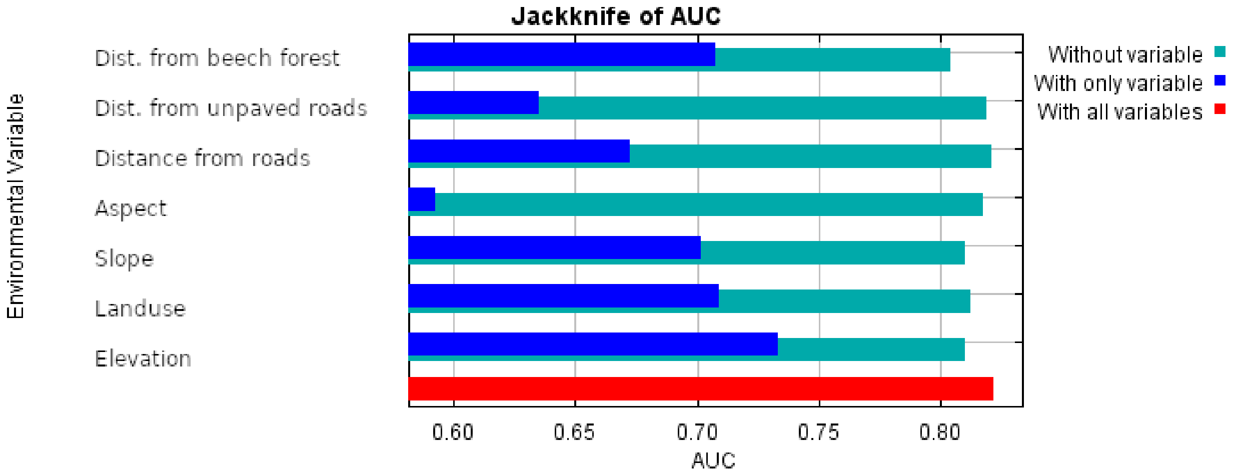

Table S1, Environmental variables used in MaxEnt or GLMM or both

Variable	Unit	Source	Original Resolution	Model
Elevation	meters	DTM LiDar Survey https://webgis.provincia.tn.it/	2 m	Both
Slope	Degrees	Derived from DTM	10 m	MaxEnt
Aspect	Degrees	Derived from DTM	10 m	MaxEnt
Terrain Ruggedness Index		Derived from DTM	10 m	GLMM
Landuse	Classes: 1. populated areas; 2. rural areas; 3. Pastures; 4. nature reserves; 5. Rocks; 6. Forests; 7. Water	https://webgis.provincia.tn.it/	10 m	MAxEnt
Distance from roads	meters, calculated from vector map	http://sdi-pat.provincia.tn.it/webgis	10 m	Both
Distance from unpaved roads roads	meters, calculated from vector map	http://sdi-pat.provincia.tn.it/webgis	10 m	Both
Distance from beech forest boundary	Calculated from vector map of forest types	https://webgis.provincia.tn.it/ (Forest types by Odasso, 2002)	10 m	Both
Cadaster map	Classes:human settlements, orchards (i.e., apples and vineyards), agriculture fields	http://www.urbanistica.provincia.tn.it/pianificazione/piano_urbanistico_provinciale/cartografia/	10 m	GLMM
